# Protection Efficacy of C5A Against Vaginal and Rectal HIV Challenges in Humanized Mice

**DOI:** 10.2174/1874357901812010001

**Published:** 2018-02-28

**Authors:** Philippe A. Gallay, Udayan Chatterji, Aaron Kirchhoff, Angel Gandarilla, Richard B. Pyles, Marc M. Baum, John A. Moss

**Affiliations:** 1 Department of Immunology & Microbiology, The Scripps Research Institute; La Jolla, California 92037, USA; 2 Department of Pediatrics, University of Texas Medical Branch; Galveston, Texas 77555-0436, USA; ^3^Department of Chemistry, Oak Crest Institute of Science; Monrovia, California 91107. USA

**Keywords:** HIV transmission, Microbicide, Bone marrow/Liver/Thymus Mice, C5A, Vaginal challenge, Rectal challenge

## Abstract

**Introduction:**

In the absence of a vaccine, there is an urgent need for the identification of effective agents that prevent HIV transmission in uninfected individuals. Non-vaccine Biomedical Prevention (nBP) methods, such as topical or systemic pre-exposure prophylaxis (PrEP), are promising strategies to slow down the spread of AIDS.

**Methods:**

In this study, we investigated the microbicidal efficacy of the viral membrane-disrupting amphipathic SWLRDIWDWICEVLSDFK peptide called C5A. We chose the bone marrow/liver/thymus (BLT) humanized mouse model as vaginal and rectal HIV transmission models.

**Results:**

We found that the topical administration of C5A offers complete protection against vaginal and rectal HIV challenges in humanized mice. After demonstrating that C5A blocks genital HIV transmission in humanized mice, we examined the molecular requirements for its microbicidal property. We found that the removal of four amino acids on either end of C5A does not diminish its microbicidal efficacy. However, the removal of four amino acids at both the ends, abolishes its capacity to prevent vaginal or rectal HIV transmission, suggesting that the length of the peptide is a critical parameter for the microbicidal activity of C5A. Moreover, we demonstrated that the amphipathicity of the helical peptide as well as its hydrophobic surface represents key factors for the microbicidal activity of C5A in humanized mice.

**Conclusion:**

With its noncellular cytotoxic activity, its property of neutralizing both HSV and HIV, and its unique mechanism of action that disrupts the stability of the viral membrane, C5A represents an attractive multipurpose microbicidal candidate to be combined with other anti-HIV agents including antiretrovirals.

## 1 INTRODUCTION

Given that there is currently no vaccine, there is an immediate need for effective agents to decrease the significant number of new HIV transmissions, recently estimated to be at ~7000 new infections/day globally [1]. Non-vaccine Biomedical Prevention (NBP) methods in the form of topical or systemic PrEP are attractive strategies to control AIDS dissemination [2-15]. Several clinical trials, based on regimens of tenofovir (TFV), frequently in combination with emtricitabine (FTC), provided evidence that PrEP could significantly reduce HIV infection in individuals [16-24].The CAPRISA 004 trial provided the first demonstration that a topical microbicide could prevent HIV transmission in humans. Specifically, a 1% tenofovir (TVF) gel used pericoitally decreased the incidence of HIV transmission in South African women by 39% [16]. However, two additional trials with 1% tenofovir gel with pericoital (VOICE) [25] and daily (FACTS) [26] dosing regimens failed to provide efficacy against new sexual HIV infections. In the ASPIRE trial of a monthly intravaginal ring delivering dapivirine, incidence of HIV infection was significantly reduced for women who wore the ring consistently, but low overall efficacy (27% risk reduction) resulted from poor adherence in certain sub-groups, particularly among younger women [27]. Indeed, poor adherence to the prophylaxis regimens may be a primary factor in this lack of efficacy; although additional factors cannot be ruled out to account for the disparate range of efficacy in the results, as indicated in a randomized pharmacokinetic crossover study [28], where the tissue concentration advantage (>100x) seen topically as compared to oral dosing was not reflected in the seroconversion data of the CAPRISA and VOICE trials. This may indicate that factors beyond the purely antiviral effect may reduce topical PrEP efficacy. Potential factors include concentration-dependent tissue toxicity from TFV, tenofovir-diphosphate (TFV-DP) effects, or dose frequency-dependent effects from the gel itself. Issues such as these may be difficult to adequately assess, and may be outside the scope of standard safety evaluation. In any case, the conflicting trial results certainly point out the multiple factors at work in the interactions between HIV and the host at the mucosal surfaces during initiation of infection, and highlight an urgent need for suitable *in vivo* model systems that can illuminate the various possible contributions to the contradictory clinical results.

The use of microbicides in studies, by the fact of their tissue-specific nature, requires the use of animal models [3, 23-25]. One animal model often used in the study of vaginal HIV transmission is the Rhesus macaque; in this case, the animals are infected with either Simian Immunodeficiency Virus (SIV) or SIV/HIV (SHIV) chimeric viruses [29-34]. However, this model has limitations, as it does not support HIV replication, as well as requiring sometimes prohibitive cost for primates whose accessibility is limited, especially with regard to the females. In addition, it faces disparities in host SIV and SHIV vulnerability since the primate colonies are outbred. Another animal model currently in use for testing of microbicidal candidates is the BLT (human Bone marrow, Liver, Thymus) mouse [35-47]. These humanized BLT mice are created by the implantation of human fetal liver and thymus tissues under the kidney capsule of an immunodeficient NOD scid gamma (NSG = NOD.Cg-*Prkdc^scid^ Il2rg^tm1Wjl^*/SzJ) mouse, followed three weeks later by the injection of autologous human fetal liver CD34+ cells (human hematopoietic stem cells, HSC). In the BLT mouse, a complete systemic reconstitution is achieved for all of the major human hematopoietic lineages, including T, B, monocyte/macrophage, dendritic, and natural killer cells. In addition, the developing T cell education occurs in the human thymic tissue. Fortuitously, for their use in the testing of microbicides, this widespread systemic and genital mucosal reconstitution with human lymphoid cells make female humanized BLT mice a useful tool to test both vaginal and rectal HIV transmission.

**Fig. (1):**
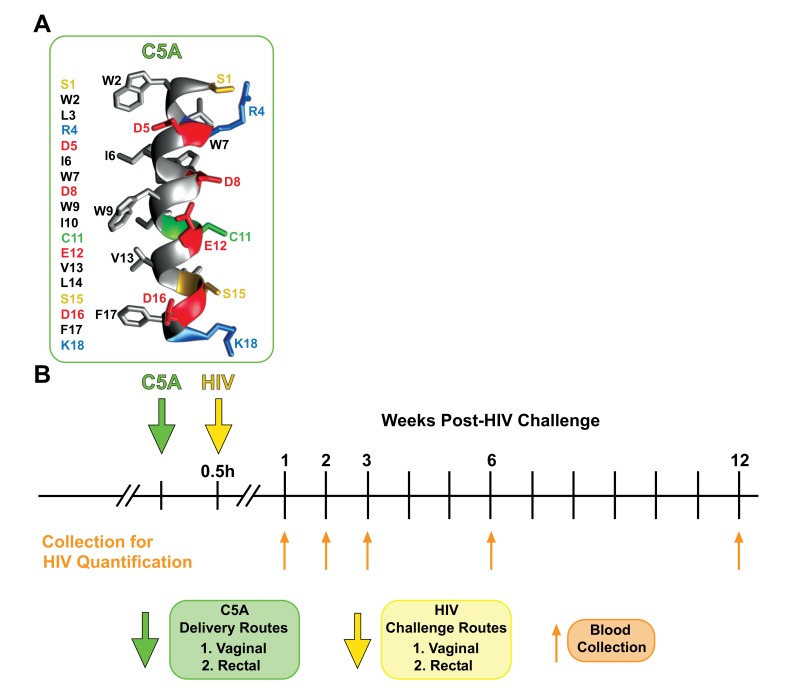
Experimental strategy for the HIV genital transmission model in humanized mice. **A.** Presented are the amino acid sequence and three-dimensional structure of the short helical peptide SWLRDIWDWICEVLSDFK C5A deduced from the nuclear magnetic resonance structure of the NS5A N-terminal membrane anchor. The image was created from structure coordinates using VMD (http://www.ks.uiuc.edu/Research/vmd/). **B.** Experimental timeline for the “humanization” of mice including the engraftment of human fetal tissues, the inoculation of autologous human CD34+ liver cells as well as the topical application of C5A prior to vaginal and rectal HIV challenges, and the viral load quantification in blood collected at the indicated time points.

The short amphipathic helical peptide SWLRDIWDWICEVLSDFK called C5A, was originally identified by the Chisari lab as an active anti-hepatitis C virus (HCV) agent [48]. The C5A amino acid sequence encompasses the region that permits the anchoring of the HCV nonstructural protein 5A (NS5A) into the ER membrane by an in-plane amphipathic alpha-helix [49, 50] (Fig. **1**). In collaboration with the Chisari lab, we demonstrated that C5A also exerts antiviral activities against HIV [51]. We found that C5A inhibits a broad range of primary HIV isolates at an nM-µM peptide concentration range and blocks infection of the three major *in vivo* targets of HIV; CD4^+^ T-lymphocytes, macrophages and dendritic cells [51]. Using an *in vitro* transwell chamber assay, which mimics HIV transmigration through primary human genital epithelial cells, we also demonstrated that C5A prevents the passage of HIV through this *in vitro* genital epithelial barrier [51]. Moreover, we showed that C5A prevents HIV transfer from Langerhans and dendritic cells to CD4^+^ T-lymphocytes [51]. Importantly, we obtained evidence for the antiviral mechanism of action of C5A. We demonstrated that C5A disrupts the membrane integrity of the HIV virion as well as the integrity of the conical capsid core that surrounds the viral genome, leading to non-infectious particles [51]. Most importantly, although C5A destabilizes the viral membrane, we showed that it preserves the integrity of cellular membranes and is not toxic to cervical epithelial cells even when used at a concentration of 10- to 100-fold greater than that which blocks HIV infection [51].

In this study, we tested the *in vivo* microbicidal efficacy of C5A against vaginal and rectal HIV challenges using the BLT mouse model. Additionally, we tested a panel of C5A variants with various degrees of anti-infectivity efficacy for their capacities to prevent vaginal and rectal HIV transmission. The main goal of this study was to investigate the microbicidal potency of C5A *in vivo* and to examine the molecular requirements of the structure and composition of C5A that determine its microbicidal activity. These data will be used to determine the most effective combination of C5A derivatives and well-characterized, small molecule ARVs with anti-HIV mechanisms of action distinct from that of C5A (*i.e*., elvitegravir, FTC or TDF), for formulation as an intravaginal ring nBP product.

## 2 MATERIAL AND METHODS

### 2.1 Creation of Humanized BLT Mice

Humanized BLT mice were created as described previously [36-38, 40, 42, 44-47, 52-54], by implanting 1-mm^3^ pieces of human fetal liver and thymus tissues (Advanced Bioscience Resources) under the kidney capsule of 6- to 8-week-old female NSG mice (Jackson Laboratories) bred at The Scripps Research Institute (TSRI). Each cohort was produced with tissues from a single donor. CD34+ hematopoietic stem and progenitor cells (HSPC) were purified from autologous fetal liver tissue, isolated by magnetic bead selection for CD34+ cells (Miltenyi), phenotyped by flow cytometry [36-38, 40, 42, 44-47, 52-54], and cryopreserved until injection (200,000-350,000 CD34+ cells) into mice 3 weeks after fetal liver and thymus tissue implantation. Human cell population reconstitution in peripheral blood was confirmed by flow cytometry as described previously [36-38, 40, 42, 44-47, 52-54]. Briefly, the degree of humanization of the BLT mice was evaluated at 20 weeks of age (10 weeks post-CD34+ HSPC injection) by analysis of percentages of human CD45+ cells and human CD45+ CD4+ CD3+ cells in peripheral blood by FACS. Mice with <65% human CD45+ cells and <70% human CD45+ CD4+ CD3+ cells were not used for HIV exposure experiments. Mice were kept at the Department of Animal Resources at TSRI in accordance with protocols approved by the TSRI Institutional Animal Care and Use Committee.

### 2.2 Vaginal and Rectal HIV Infection Studies in Humanized BLT Mice

The parent and modified C5A peptides were obtained from GenScript, and their amino acid sequences are given in Fig. (**1A**) and Table **1**. Solutions of C5A peptides were prepared in phosphate buffered saline (PBS). After verifying the reconstitution of mice with human cells, a dose ranging study was conducted to determine the EC_50_ of C5A for preventing vaginal and rectal HIV infection. Stocks of HIV JR-CSF were prepared as previously described [46, 47] and standardized by p24 ELISA. Prior to inoculation, mice were anesthetized with isoflurane. Aliquots of C5A in PBS (5 µL) were administered vaginally or rectally in decreasing concentrations (200, 100, 50, 25, 12.5 and 6.25 µM; n=10 mice per concentration). Fifteen minutes post-C5A topical administration, mice were challenged (vaginal challenge for vaginal C5A administration, rectal challenge for C5A rectal administration) with HIV. Atraumatic vaginal and rectal HIV inoculations were conducted as previously described [46, 47] using a total volume of 5 µL (200 ng of p24 - 20,000 of 50% tissue culture infective dose (TCID__50%__) (Fig. **1B**). Infection was quantified by measuring levels of viral RNA in peripheral blood at weeks 1, 2, 3, 6 and 12. Infection studies comparing the modified C5A peptides to the C5A parent were conducted using an identical procedure, with vaginal or rectal application of a single 100 µM peptide dose (n=10 mice per peptide group) 15 min prior to vaginal or rectal HIV challenge, respectively. Note that upon viral genome sequencing, viruses that replicate in the peripheral blood of mice did not differ from the inoculated JR-CSF (data not shown).

### 2.3 Viral Load Quantification in Humanized BLT Mice

Infection of BLT mice was analyzed by quantifying HIV RNA levels in peripheral blood (plasma) using one-step reverse transcriptase quantitative real-time PCR (ABI custom TaqMan Assays-by-Design) according to the manufacturer’s instructions. Primers were 5-CATGTTTTCAGCATTATCAGAAGGA-3 and 5-TGCTTGATGT CCCCCCACT-3, and MGB-probe 5-FAM-CCACCCCACAAGATTTAAACACCATGCTAA-Q-3, where FAM is 6-carboxyfluorescein [36-38, 40, 42, 44-47, 52-54]. The assay sensitivity was of 300-500 400 RNA copies per mL.

### 2.4 Data Analysis

Data were analyzed using GraphPad Prism (version 7.00, GraphPad Software, Inc., La Jolla, CA). Analytic simulations of dose-response curves using the median-effect principle and mass-action law, and its combination index theorem [55] were carried out using CompuSyn [56].

## 3 RESULTS

### 3.1 Dose-ranging Study to Evaluate C5A Efficacy Against Vaginal and Rectal HIV Challenges in BLT Mice

The amounts of topically applied C5A required to block HIV transmission in BLT mice challenged either vaginally or rectally were evaluated in a dose-ranging infection study. Vaginally applied C5A offered no protection against a vaginal HIV challenge at 6.25 and 12.5 µM, 30% protection at 25 µM, 80% protection at 50 µM and 100% protection at 100 and 200 µM (Table **2**). Similar protection results were obtained for the rectal challenge of HIV: no protection at 6.25 and 12.5 µM, 50% protection at 25 µM, 80% protection at 50 µM and 100% protection at 100 and 200 µM (Table **2** and Appendix. **A**). The absence of HIV RNA in blood correlated well with the absence of HIV DNA in tissues including spleen, lymph node, thymic organoid, liver, lung and female reproductive tract (data not shown). Dose-response curves for vaginal and rectal HIV prevention efficacy by C5A are shown in Fig. (**2**), along with fits of the data to a sigmoidal dose-response (variable slope) model. From the model, EC_50_ values of 32.5 and 25.7 μM were calculated for vaginal and rectal dosing, respectively.

### 3.2 Effect of Amino Acid Sequence Modifications on C5A Microbicidal Properties

The protection efficacy of C5A was compared to efficacy of modified C5A peptides with C-terminal (C5A-C), N-terminal (C5A-N), or C- and N-terminal truncations (C5A-CN). For 100 µM C5A (18 amino acid length), C5A-C (14 amino acid length), or C5A-N (14 amino acid length), 100% protective efficacy was maintained against a vaginal or a rectal HIV challenge (Table **3** and Appendix. **B**). In sharp contrast, C5A-CN (10 amino acid length) with both ends truncated offered no protection (Table **3**). Both the non-amphipathic (C5A-NA) and hydrophobicity-scrambled (C5A-HS) variants show no protection against vaginal or rectal challenge by HIV (Table **3** and Appendix. **C**), suggesting that the amphipathic and hydrophobic nature of C5A is required for its ability to block vaginal and rectal HIV transmission in humanized BLT mice.

## 4 DISCUSSION AND CONCLUSION

**Fig. (2):**
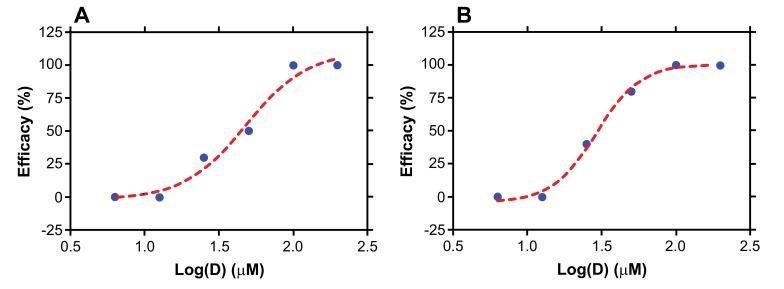
Calculation of EC_50_ of C5A providing protection against (A) vaginal and (B) rectal HIV challenges in humanized BLT mice.

In the absence of a safe and effective vaccine, there is an imperative demand for effective agents to reduce the 7,000 new HIV infections occurring every day. An attractive possibility is to identify the best drugs and drug combinations, delivered by optimal nBP methods such Intravaginal Rings (IVRs) for vaginal delivery and gels or enemas for rectal protection. We previously reported that C5A, a short, amphipathic, 18 amino acid (SWLRDIWDWICEVLSDFK) helical peptide, inhibits *in vitro* infectivity of a broad range of primary HIV isolates in various primary target cells [51]. In this study, we evaluated the microbicidal efficacy of C5A *in vivo* in a humanized mouse model, and investigated the structural requirements of C5A to block HIV transmission *in vivo*. These studies show that parental C5A efficiently inhibits both vaginal and rectal HIV transmission in humanized mice. This is in accordance with a previous study demonstrating that topical vaginal administration of 200 µM C5A in PBS confers 100% protection against a vaginal challenge of HIV in humanized mice [38]. This is also in accordance with our previous study that showed that C5A maintains its antiviral activities in genital fluids including seminal plasma, cervical and vaginal fluids [57]. Moreover, this is in agreement with our recent study that showed that a vaginal application of C5A protects 89% of macaques from a simian-human immunodeficiency virus (SHIV-162P3) challenge [58]. Although the vaginal pH varies between species – 3.5 to 4.5 for humans and 6 to 7 for mice and macaques is 6 to 7 [59, 60] - we previously reported that C5A keeps is antiviral activity between pH 5 and 7, but loses its activity at pH 8 [51]. For the humanized mouse studies reported here, 50% effective concentrations (EC_50_) of C5A calculated using the traditional sigmodal dose-response model were 32.5 and 25.7 µM, respectively, for vaginal and rectal HIV challenge (Fig. **2**). A major advantage of the humanized mouse model is the ability to create large groups of animals from tissues from a single donor. We routinely generate 60-80 humanized mice per liver/thymus tissue. Another advantage of this model is the capacity of using high numbers of animals per group/treatment (n = 10) to attain a satisfactory statistical power. This further confirms that the BLT mouse model is an ideal tool to screen microbicidal candidates.

**Fig. (3):**
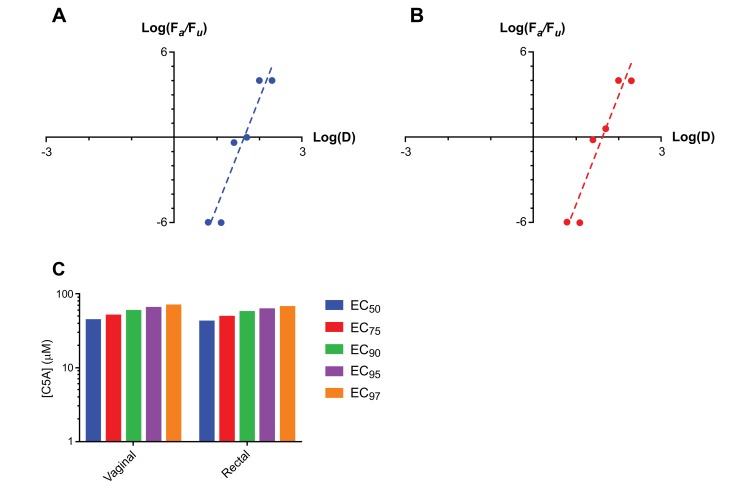
Median-effect plots describing the impact of (A) vaginal (regression coefficient, *r* = 0.956) and (B) rectal (regression coefficient, *r* = 0.955) C5A dosing on HIV preventions in humanized BLT mice. The model allows a range (50-97%) of C5A effective concentrations to be calculated for both compartments (C).

Applying the median-effect model to the humanized mouse dose-ranging efficacy datasets allowed EC values in the 50-97% protection range to be calculated (Fig. **3**). The slope, *m,* of the linear fit to the log-log median-effects plots Figs. (**3A** and **3B**) is analogous to the Hill coefficient and provides a quantitative estimation of the sigmoidicity of the dose-effect curve. Slope parameters (*m*) for C5A of 7.6 ± 1.2 and 7.7 ± 1.2 were calculated for the vaginal and rectal compartments, respectively. Fig. (**3**) summarizes the results from this expanded analsyis, where *F_a_* represents the fraction affected and *F_u_* the fraction unaffected. The high regression coefficients (*r* > 0.95) support the applicability of the model. Shen *et al*. used an *ex vivo* HIV model to show that ARV agents has a characteristic slope, ranging from 1 for nucleoside reverse transcriptase inhibitors (NRTIs) to 1.8-4.5 protease inhibitors (PIs) [61]. The much higher slopes (*m*) of the C5A dose-response curves (7.6 and 7.7 for vaginal and rectal dosing, respectively) observed *in vivo* suggest that this microbicide has significant potential as an efficatious PrEP agent, as illustrated in Fig. (**3C**). To our knowledge, this is the first demonstration that the humanized BLT mouse model allows the determination of ECs of a drug against vaginal or rectal HIV transmission.

We found that the genital administration of 25 or 50 µM of C5A provides only a partial protection. We believe that these concentrations of the virucidal peptide in the genital compartment do not neutralize (rupture) 100% of incoming viruses, allowing some infectious particles to cross the genital epithelium and to reach the peripheral blood where CD4+ T lymphocytes will be infected and will support robust HIV replication.

The studies with modified C5A-C and C5A-N demonstrate that the removal of four amino acids at either end of C5A does not alter the microbicidal efficacy of the peptide. In sharp contrast, the simultaneous removal of four amino acids at each end (C5A-CN) abolishes the microbicidal capabilities of C5A, suggesting that the peptide requires a sufficient length to effectively block both vaginal and rectal HIV transmissions.

The lack of protection observed for C5A-NA demonstrates that the amphipathic structural nature of C5A is critical for its microbicidal properties. Similarly, C5A-HS offered no protection vaginally or rectally, indicating that the hydrophobic face of C5A (or NS5A), thought to mediate anchoring into the ER membrane, is also critical for the ability of the peptide to inhibit HIV infection.

Following these studies with unformulated C5A solutions applied topically, the next goal is to formulate C5A, alone and in combination with other antiretroviral agents, into murine intravaginal rings to evaluate sustained topical delivery approaches for protection against vaginal and rectal HIV challenges. We plan on using a combination of drugs with distinct mechanisms of action. Remarkably, C5A possesses a unique anti-HIV mechanism of action. We demonstrated that C5A disrupts the stability of the membrane of HIV virions as well as that of the capsid core, surrounding the viral genome, resulting into non-infectious particles [51]. Our working hypothesis is that anchoring C5A molecules *via* their hydrophobic surface into the HIV membrane, destabilizes and ruptures the integrity of the viral membrane. This means that C5A theoretically would act in the lumen (vaginal or rectal), much like the traditional microbicides. This mode of action would be highly complementary to small-molecule ARV PrEP candidates (e.g., TDF and FTC) as these inhibit viral replication in immune cells, presumably in tissues (vaginal or rectal), a different anatomic compartment. Importantly, C5A does not alter the cellular membranes even when used at a concentration of 10- to 100-fold greater than that which blocks HIV infection [51]. This is in accordance with our recent study that demonstrated that a vaginal application of C5A protects 89% of macaques from a simian-human immunodeficiency virus (SHIV-162P3) challenge and that no signs of lesions or inflammation were observed in animals vaginally treated with repeated C5A applications [57]. With its noncellular cytotoxic activity and rare mechanism of action, C5A represents an attractive microbicidal candidate to be combined with other drugs, including ARVs, with different antiviral mechanisms of action.

An additional attractive property that C5A offers is that it also efficiently inhibits HSV infection [58], potentially acting as a single-agent multipurpose protection technology (MPT). Previous studies have shown that C5A inhibits HSV infection of Vero cells, human dendritic cells and primary genital epithelial cells *in vitro* as well as human epidermis (epidermal sheets) *ex vivo*, and that it inhibits acyclovir- and ganciclovir-resistant HSV isolates [58]. Remarkably, in an *in vitro* transwell assay, C5A prevented HIV transmigration by preserving the integrity of the genital epithelium that is normally severely compromised by HSV pre-infection [58]. Because genital herpes is a major risk factor in acquiring HIV infection, C5A represents a multipurpose microbicide candidate, which may interfere with HIV transmission via a dual mechanism of action – neutralizing both HSV and HIV.

## Figures and Tables

**APPENDIX A:**
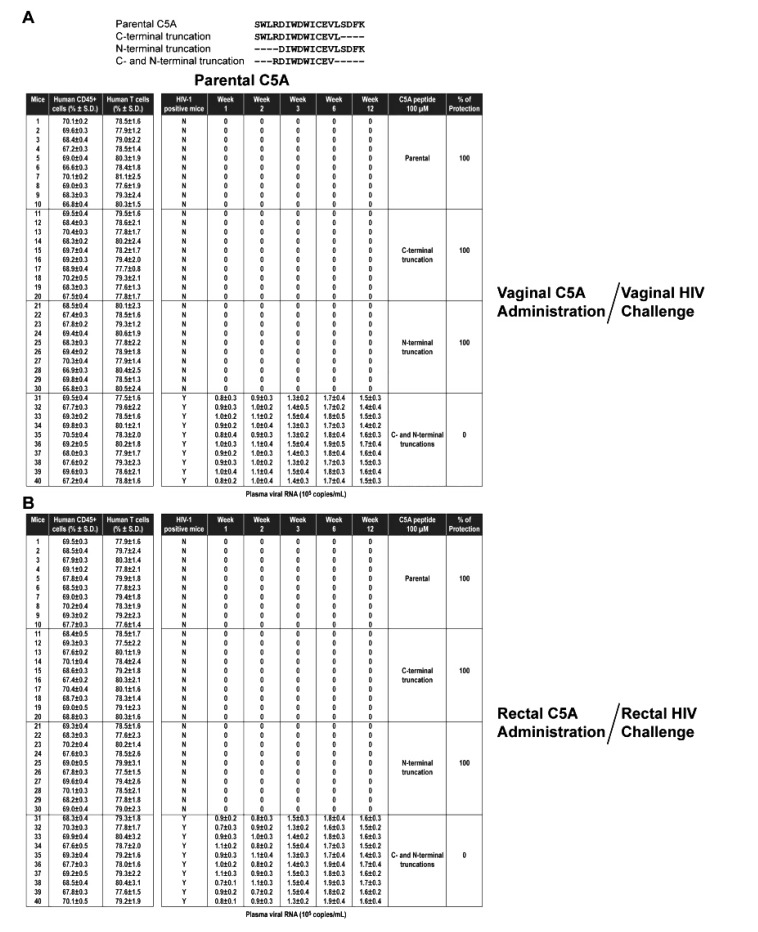
Protection by topical administration of parental C5A against vaginal and rectal HIV challenges in humanized mice. On the left the numbers of mice per treatment are indicated (n = 10) as well as the percentages of human CD45+ and CD45+ CD4+ CD3+ cells. In the middle are the numbers of 10^5^ copies per mL of plasma collected at weeks 1, 2, 3, 6 and 12. On the right are the concentrations of parental C5A SWLRDIWDWICEVLSDFK applied vaginally (A) or rectally (B) 15 min prior to a vaginal (A) and rectal (B) HIV (JR-CSF) challenge.

**APPENDIX B:**
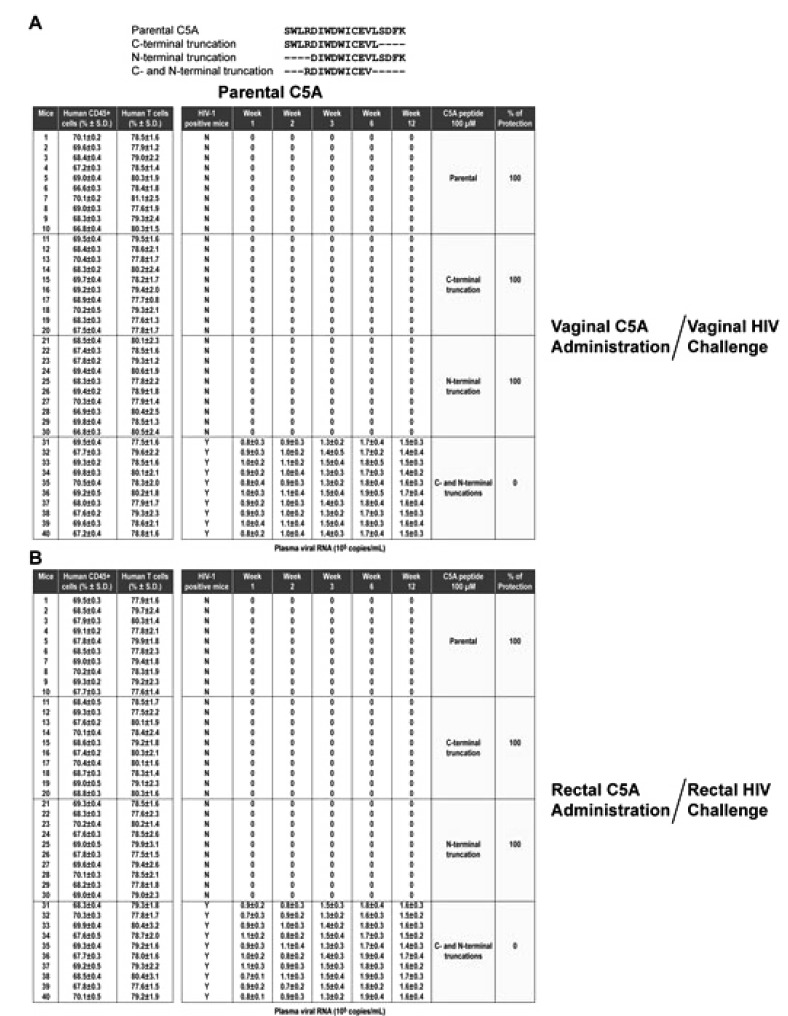
The removal of both ends of C5A abrogates its capacity to inhibit vaginal and rectal HIV transmission in humanized mice.Same as Appendix.(**A**) except that indicated on the right are the amino acid sequences of the C5A peptides (100 µM) applied vaginally (A) or rectally (B) 15 min prior to a vaginal (A) and rectal (B) HIV (JR-CSF) challenge.

**APPENDIX C:**
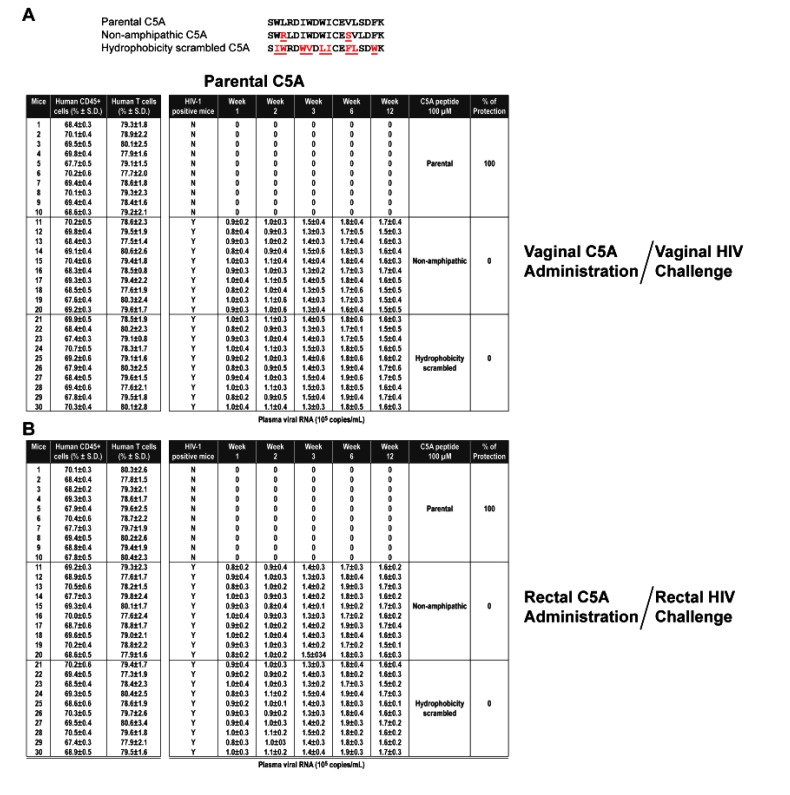
The amphipathic and hydrophobic nature of C5A controls the ability of the short peptide to prevent vaginal and rectal HIV transmission in humanized mice. Same as Appendix.(**A**) except that indicated on the right are the amino acid sequences of the C5A peptides (100 µM) applied vaginally (A) or rectally (B) 15 min prior to a vaginal (A) and rectal (B) HIV (JR-CSF) challenge.

**Table 1:** Nomenclature and amino acid sequence for Parent and Modified C5A.

**Abbrev.**	**Description**	**Sequence**	**Sequence Length**
**C5A**	C5A Parent	SWLRDIWDWICEVLSDFK	18 AA
**C5A-C**	C-terminal truncation	SWLRDIWDWICEVL––––	14 AA
**C5A-N**	N-terminal truncation	––––DIWDWICEVLSDFK	14 AA
**C5A-CN**	C- and N-terminal truncation	–––RDIWDWICEV–––––	10 AA
**C5A-NA**	Non-amphipathic C5A	SWRLDIWDWICESVLDFK	18 AA
**C5A-HS**	Hydrophobicity-scrambled C5A	SIWRDWVDLICEFLSDWK	18 AA

**Table 2:** Protection by topical administration of C5A against vaginal and rectal HIV challenges.

**C5A Conc. (μM)**	**% Protection (n=10)**	
	**Vaginal**	**Rectal**
**6.25**	0% (0/10)	0% (0/10)
**12.5**	0% (0/10)	0% (0/10)
**25**	30% (3/10)	50% (5/10)
**50**	80% (8/10)	80% (8/10)
**100**	100% (10/10)	100% (10/10)
**200**	100% (10/10)	100% (10/10)

**Table 3:** Effect of amino acid sequence modifications on capacity of C5A to inhibit vaginal and rectal HIV transmission.

**C5A Variant (100 μM)**	**% Protection (n=10)**	
	**Vaginal**	**Rectal**
**C5A**	100% (10/10)	100% (10/10)
**C5A-C**	100% (10/10)	100% (10/10)
**C5A-N**	100% (10/10)	100% (10/10)
**C5A-CN**	0% (0/10)	0% (0/10)
**C5A-NA**	0% (0/10)	0% (0/10)
**C5A-HS**	0% (0/10)	0% (0/10)
